# Ultrasensitive Nonenzymatic Real-Time Hydrogen Peroxide Monitoring Using Gold Nanoparticle-Decorated Titanium Dioxide Nanotube Electrodes

**DOI:** 10.3390/bios13070671

**Published:** 2023-06-22

**Authors:** Md. Ashraful Kader, Nina Suhaity Azmi, A. K. M. Kafi, Md. Sanower Hossain, Rajan Jose, Khang Wen Goh

**Affiliations:** 1 Faculty of Industrial Sciences and Technology, Universiti Malaysia Pahang, Kuantan 26300, Malaysia; 2 Department of Chemistry and Biochemistry, Kent State University, Kent, OH 44242, USA; 3 Centre for Sustainability of Ecosystem and Earth Resources (PUSAT ALAM), Universiti Malaysia Pahang, Kuantan 26300, Malaysia; 4 Center for Advanced Intelligent Materials, Universiti Malaysia Pahang, Kuantan 26300, Malaysia; 5 Faculty of Data Science and Information Technology, INTI International University, Nilai 71800, Malaysia

**Keywords:** amperometry, electrochemical H_2_O_2_ sensor, gold nanoparticles, nonenzymatic detection, titanium dioxide nanotube

## Abstract

An amperometric enzyme-free hydrogen peroxide (H_2_O_2_) sensor was developed by catalytically stabilizing active gold nanoparticles (Au NPs) of 4–5 nm on a porous titanium dioxide nanotube (TiO_2_ NTs) electrode. The Au NPs were homogeneously distributed on anatase TiO_2_ NTs with an outer diameter of ~102 nm, an inner diameter of ~60 nm, and a wall of thickness of ~40 nm. The cyclic voltammogram of the composite electrode showed a pair of redox peaks characterizing the electrocatalytic reduction of H_2_O_2_. The entrapping of Au NPs on TiO_2_ NTs prevented aggregation and facilitated good electrical conductivity and electron transfer rate, thus generating a wide linear range, a low detection limit of ~104 nM, and high sensitivity of ~519 µA/mM, as well as excellent selectivity, reproducibility, repeatability, and stability over 60 days. Furthermore, excellent recovery and relative standard deviation (RSD) were achieved in real samples, which were tap water, milk, and *Lactobacillus plantarum* bacteria, thereby verifying the accuracy and potentiality of the developed nonenzymatic sensor.

## 1. Introduction

Hydrogen peroxide (H_2_O_2_) is a multifunctional chemical that acts as an oxidizing agent in a variety of industrial environments [1] and as a signal messenger in mediating cellular processes [2]. H_2_O_2_ is utilized to disinfect food industry equipment used in the mixing, bottling, transport, and packing processes [3]. As an antibacterial agent, H_2_O_2_ is used to preserve milk and juice [4]. Generally, a large quantity of H_2_O_2_ at high concentrations (usually 35% and above) is used in industrial applications and might cause toxicity regardless of the exposure routes. Exposure to high concentrations of H_2_O_2_ preferentially induces necrosis, and moderate concentrations can cause apoptosis. Additionally, H_2_O_2_ is very stable and can rSeach diverse molecular targets far from the origin of generation [5].

H_2_O_2_ is produced by some bacteria, such as *Lactobacillus plantarum,* used in the food processing industry. *L. plantarum* is a microbial starter and probiotic that can produce H_2_O_2_ as well as other compounds, including organic acids and diacetyl [6]. Hence, the presence of H_2_O_2_ in food at an intolerable concentration for the human body could pose a threat to consumer health. Moreover, exogenously generated H_2_O_2_ can induce DNA damage, ATP depletion, apoptosis, necrosis, and severe cytotoxicity [7]. Therefore, precise and sensitive detection of H_2_O_2_, especially at the micro and nano levels, is required to ensure healthy lives and to promote well-being as stated in the sustainable development goal (SDG#3) adopted by the United Nations.

To date, chemiluminescence, spectrophotometry, chromatography, and electrochemical sensors have all been developed to detect H_2_O_2_ [8]. However, the electrochemical sensors have gained prominence due to their simplicity, sensitivity, selectivity, and low-detection capability [9]. More precisely, amperometry-based electrochemical sensors were developed using highly catalytically active horseradish peroxidase (HRP) and hemoglobin (Hb) [10]. However, complex immobilization, H_2_O_2_-induced inactivation of proteins, the high cost of enzymes, and their sensitivity to environmental conditions have limited the use of these molecules in sensor development [11,12].

Currently, nanomaterials are widely used in electrochemical H_2_O_2_ sensor fabrication, which has overcome the bottlenecks of HRP and Hb-modified sensors with high sensitivity, and are now leading the next generation of electrochemical sensors [13,14,15]. Plasmonic nanostructures such as gold (Au NPs), platinum (Pt NPs), and silver nanoparticles (Ag NPs) are frequently used for H_2_O_2_ sensing [16]. In particular, Au NPs are used for various chemical and biomolecule detections due to their desirable biocompatibility, large specific surface area, high extinction coefficients, and excellent conductivity [8]. Au NPs have excellent nanozyme activities resembling peroxidase, oxidase, catalase, and reductase [17]. This enzyme-mimicking property can promote electron transfer through the interface of Au NPs and expand the outer region of the modified electrode during sensing [18]. It has also been reported that nanomaterials with enzyme-like properties have the potential to overcome the intrinsic limitations of natural enzymes such as low stability and storage difficulties [19]. 

The high surface-to-volume ratio of Au NPs provides superior catalytic efficiency; however, it unfavorably reduces chemical stability and causes aggregation [20]. Moreover, the tiring and time-consuming re-dispersion cycle affects the performance of Au NPs [21]. Entrapment of Au NPs within a porous structure is a very sustainable approach, as the porous material can confine the metal nanoparticles and prevent aggregation. In addition, porous materials exhibit a size-selective property that ensures accurate interaction of the reactants with the metal surface [22].

Many metal oxides, such as copper(II) oxide (CuO), titanium dioxide(TiO_2_), manganese(IV) oxide (MnO_2_), zinc oxide (ZnO), tungsten trioxide (WO_3_), and tin(iv) oxide (SnO_2_) can be synthesized as porous structures with different shapes, such as nanotubes (NTs) [23,24]. TiO_2_ NTs have desired properties including a tubular structure, larger aspect ratio, corrosion resistance, biocompatibility, high chemical and thermal stability, non-toxicity, and chemical inertness, making them a suitable choice for developing Au NPs-TiO_2_ NTs composite sensors [25,26,27]. The TiO_2_ NTs synthesized via anodization offer a porous structure [28]. The tubular inner pores of TiO_2_ NTs help to effectively entrap platinum (Pt), palladium (Pd), and Au nanoparticles in their hollow structure and improve catalytic performance [29,30]. This entrapment also inhibits electron-hole pair recombination to achieve high charge transfer efficiency and catalytic activity [31]. In addition, TiO_2_ NTs-supported metals show better catalytic performance than carbon-based nanostructures [32]. To coat Au NPs on these porous TiO_2_ NTs, a weakly conductive chitosan (CS) polymer is reported to protect the electrode material without affecting the catalytic performance [33]. 

This study developed a simple, highly susceptible Au NPs-TiO_2_ NTs composite sensor by decorating Au NPs on TiO_2_ NTs for the real-time monitoring of H_2_O_2_. The nanostructure and morphology of the Au NPs-TiO_2_ NTs composite were characterized using field-emission scanning electron microscopy (FESEM) and X-ray powder diffraction (XRD). The electrochemical property and H_2_O_2_-sensing performances were evaluated using cyclic voltammetry and multi-step chronoamperometry.

## 2. Materials and Methods

### 2.1. Reagents and Materials

Titanium (Ti) foil, dimethyl sulfoxide (DMSO), chloroauric acid hydrate (HAuCl_4_·H_2_O), sodium citrate (Na_3_C_6_H_5_O_7_), sodium borohydride (NaBH_4_, 98%), chitosan (crab shells), acetic acid (CH_3_COOH, 99%), sodium phosphate monobasic (NaH_2_PO_4_, 99%) and sodium phosphate dibasic (Na_2_HPO_4_, 99%), sodium hydroxide (NaOH) pellet, hydrochloric acid (HCl), and hydrogen peroxide (H_2_O_2_, 30 wt%) were purchased from Sigma-Aldrich (St. Louis, MI, USA). Hydrofluoric acid (HF, 49%) was purchased from Fisher Chemical (Waltham, MA, USA). The Difco™ Lactobacilli MRS Agar and *Lactobacilli* MRS broth were purchased from Merck Millipore (Darmstadt, Germany). H_2_O_2_ was preserved at 4 °C. Ultrapure water (18 MΩ·cm) purified with a Nanopure^®^ water system (Merck, Germany) was used to prepare all experiment solutions. All the reagents were of analytical grade.

### 2.2. Preparation of the Au NPs

The Au NPs used in this work were prepared following the citrate reduction method [34]. Briefly, first of all, 1 mL of 1% (*w*/*w*) sodium citrate solution was added to 100 mL of 0.01% (*w*/*w*) HAuCl_4_ aqueous solution at room temperature under continuous stirring. Then, after 1 min, 1.6 mL of 0.075% (*w*/*w*) NaBH_4_ that was prepared in the 1% (*w*/*w*) sodium citrate solution was added to the solution slowly and stirred continuously until its color turned red. The synthesized Au NPs were stored at 4 °C until further use.

### 2.3. Synthesis of TiO_2_ Nanotubes 

The TiO_2_ nanotubes used in this study were synthesized using an anodic oxidation method [35,36]. Before the anodic oxidation, titanium (Ti) foil (0.8 × 1.0 × 0.05 cm) was cleaned with acetone and ethanol by ultrasonic treatment. The Ti foil was then washed with distilled water and etched in 18% HCl (*v*/*v*) solution for 10 min at 85 °C. After etching, the Ti foil was cleaned with ultrapure water. It was then used as the working electrode for anodic oxidation in a two-electrode electrochemical cell, where a platinum coil was used as the counter electrode. The anodic oxidation of Ti foil was performed by applying a voltage of 40 V for 8 h in an electrolyte containing DMSO and HF (2%). After anodization, the synthesized TiO_2_ NTs were rinsed with ultrapure water and subsequently ultrasonicated to remove surface residues. Finally, the TiO_2_ nanotubes were annealed at 450 °C for 1 h in an ambient atmospheric condition to enhance the crystalline properties and to remove remnants.

### 2.4. Fabrication of Au NPs-TiO_2_ NTs Composite Electrode

The Au NPs-TiO_2_ NTs composite electrode was prepared by direct casting of Au NPs onto the TiO_2_ nanotubes. Before casting the Au NPs, the prepared TiO_2_ NTs were cleaned using ethanol and ultrapure water for 5 min and dried in air. Then, 16 µL of Au NPs was immobilized on the TiO_2_ NTs surface with 9 µL of chitosan (2 mg/mL) and dried in air. These composites were used as a working electrode for further analysis. 

### 2.5. Morphological Characterization and Electrochemical Measurement

The morphology of the Au NPs was studied using transmission electron microscopy (TEM) (Technai 20, FEI, Hillsboro, OR, USA). Field-emission scanning electron microscopy (FESEM) of TiO_2_ NTs and Au NPs-TiO_2_ NTs composite was performed using JSM-7800F (JEOL, Tokyo, Japan) at an acceleration voltage of 5 kV and an Energy Dispersive X-Ray (EDX) spectrometer. XRD patterns were acquired using an X-ray diffractometer (Miniflex II; Rigaku, Japan) by Cu-Kα radiation in the range of 2θ = 20°–70°. Electrochemical measurements were performed using a three-electrode configuration with the Gamry Potentiostat Instrument (INTERFACE1000E; 09218, UK). An Ag/AgCl (KCl saturated) electrode and a platinum wire electrode were used as the reference and counter electrode, respectively. For cyclic voltammetry (CV), the working electrode was cycled by applying a voltage of −0.1 V to 0.5 V at a scan rate of 10 mV/s. Multi-step chronoamperometry was carried out in a stirred cell by applying a potential of −0.35 V to the working electrode. All measurements were performed at room temperature with freshly prepared solutions.

### 2.6. Preparation and Analysis of Real Samples

The H_2_O_2_-sensing performance of the Au NPs-TiO_2_ NTs composite sensor in real samples was explored through multi-step chronoamperometry, by applying a potential of −0.35 V to the working electrode. To assess potency, tap water, full-cream milk, and two different sources (*tapai* and pickle) of *L. plantarum* bacteria were prepared. To evaluate the performance of H_2_O_2_ sensing in the tap water, 19 mL of tap water was added to 31 mL of PBS, and multi-step chronoamperometry was performed in this electrolyte. To perform the multi-step chronoamperometry with full-cream milk, 4 mL of commercial full-cream milk was added directly to 46 mL of PBS. 

This study used *L. plantarum* to evaluate the effectiveness of the H_2_O_2_ biosensor. The isolated and pure *L. plantarum* broth culture was given by Glycobio International Sdn. Bhd., Malaysia. Then, the test sample was prepared from a two-day-old *L. plantarum* broth culture. The two-day-old *L. plantarum* broth culture (50 mL) was centrifuged at 4000× *g* for 20 min using an ultracentrifuge. The pellet was further centrifuged at 4000× *g* for 10 min, and then the collected pellet was added to 10 mL of PBS solution (pH 7.0), and this was our test sample. During the multi-step chronoamperometry, 1 mL bacteria test sample was added to 49 mL 0.1 M PBS (pH 7.0) and left for 15 min under continuous stirring for incubation. After incubation, multi-step chronoamperometry was carried out, and 10 µM H_2_O_2_ was added at 50 s intervals up to 300 s.

The changes in the reduction response were monitored, and the recovery of H_2_O_2_ from the solution was calculated by comparison with the standard (only 0.1 M PBS solution) amperometric curve of PBS.

## 3. Results and Discussion

### 3.1. Morphological Characterization of Au NPs and TiO_2_ Nanotubes

**Figure 1:**
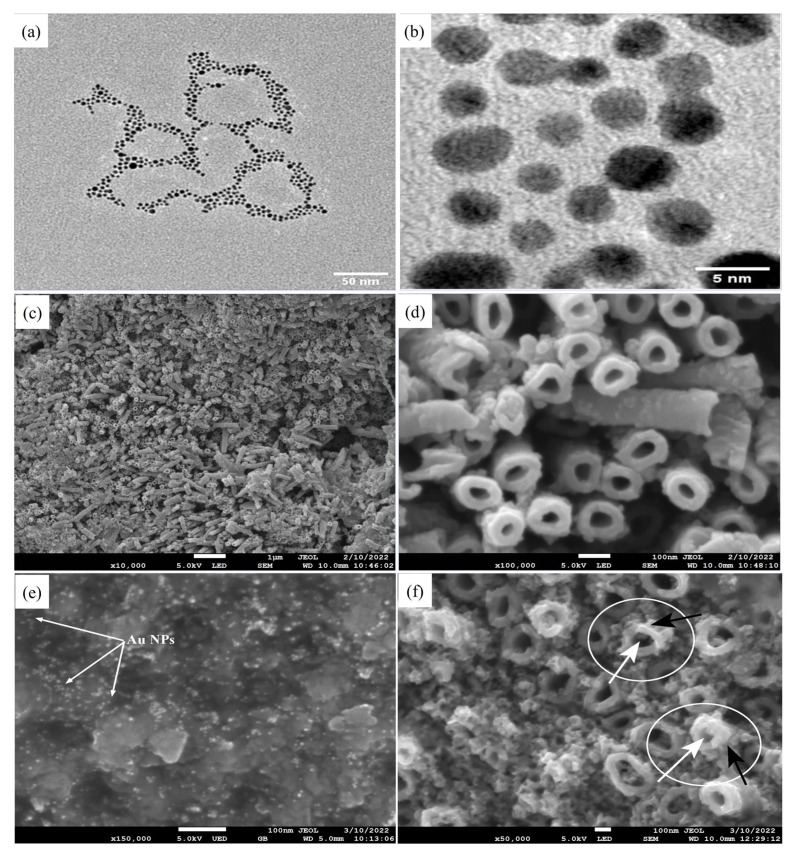
Micrographs of different microscopes show the morphology and structure of the composites of Au NPs-TiO_2_ NTs. TEM images of Au NPs (**a**) low (×29,000) and (**b**) high magnifications (×240,000); FESEM images of TiO_2_ nanotubes. (**c**) top view (×1000) and (**d**) magnified top view (×10,000); FESEM Images of Au NPs-TiO_2_ NTs (**e**) ×15,000 (White arrows shows Au NPs), and (**f**) ×50,000 magnifications (White arrow = TiO_2_, Black arrow = Au NPs).

The particle size, shape, and distribution of Au NPs were examined using TEM. Figure 1a,b show the micrographs of spherical shape Au NPs with an average particle size of 4–5 nm. In the case of Au NPs, small-sized particles exhibited much higher catalytic activity than larger ones [37]. UV-Vis spectroscopy showed an absorption peak at 519 nm (data not shown here), indicating Au NPs formation.

The structural properties of porous TiO_2_ NTs grown on a Ti substrate were studied by FESEM. The top-view images presented in Figure 1c,d show that vertically oriented TiO_2_ NTs had open-mouth structures with an outer diameter of ~102 nm, an inner diameter (pore size of NTs) of ~60 nm, a wall of thickness of ~40 nm, and an average length of ~3 µm.

### 3.2. Morphological and Structural Studies of Au NPs-TiO_2_ NTs Composite

The Au NPs-TiO_2_ NTs composite electrode prepared by coating Au NPs on the TiO_2_ NTs surface was examined by FESEM and XRD. The presence of Au NPs on the top, inner, and outer surfaces of the nanotube walls are shown in Figure 1e,f. Nano spots (white color) indicated by the arrows in Figure 1e confirmed the presence of Au NPs on the surface of TiO_2_ NTs. Moreover, the area of Au NPs (black arrow) around TiO_2_ NTs (white arrow) is marked in Figure 1f. This composite property analysis revealed open-top characteristics where both materials were in their original structure.

**Figure 2:**
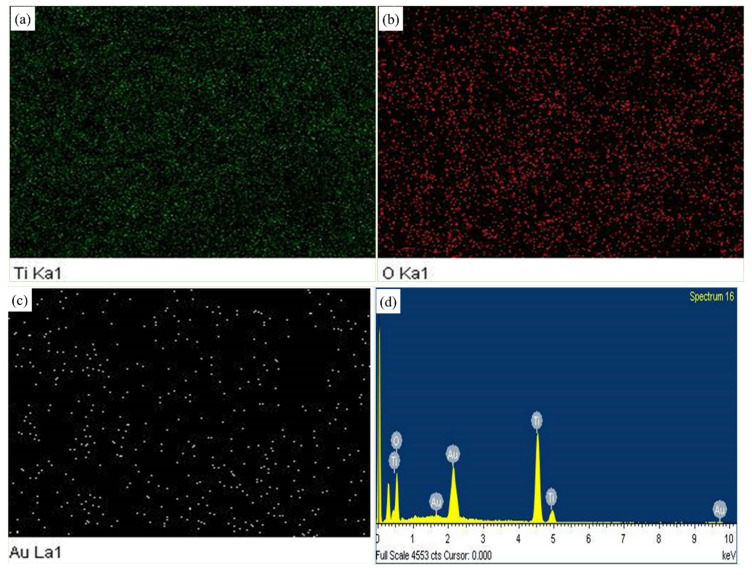
Elemental mapping images of (**a**) Ti, (**b**) O, (**c**) Au, and (**d**) EDX spectrum of Au NPs-TiO_2_ NTs.

The elemental mapping displayed in Figure 2a–c revealed the homogeneity of the deposited Au NPs along with Ti and O. The further EDX spectrum displayed in Figure 2d revealed the successful deposition of Au NPs. Au signal indicated Au NPs, and Ti and O signals represented TiO_2_ particles. Au NPs showed low intensity in EDX due to the small amount of casting suitable for nanocomposite [38].

**Figure 3:**
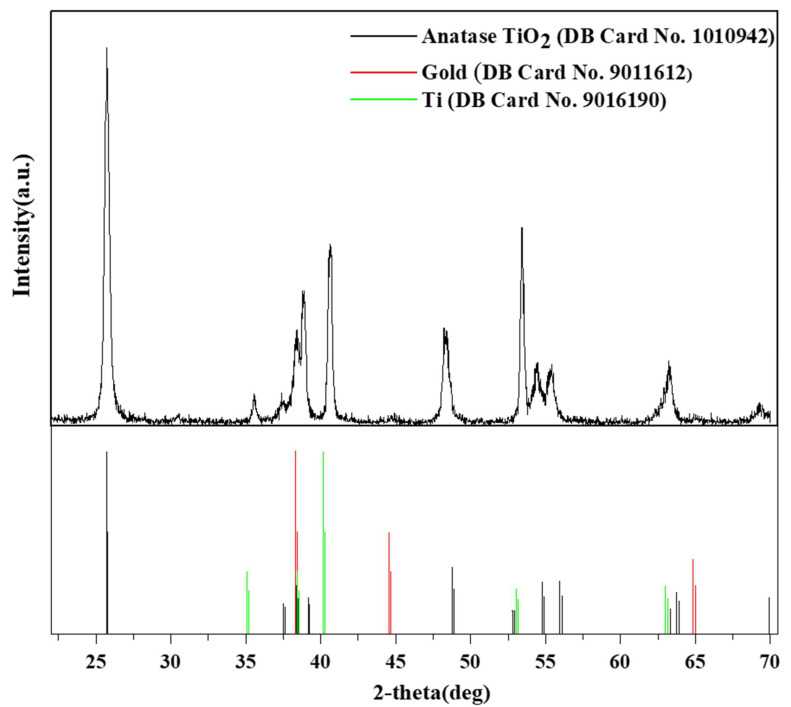
X-ray diffraction (XRD) spectrum of Au NPs-TiO_2_ NTs (up) along with anatase TiO _2_, gold and Ti (below). DB: database.

The formation of Au NPs-TiO_2_ NTs composite and its elements was further studied using XRD, as displayed in Figure 3. Since the TiO_2_ NTs were synthesized on a Ti foil and TiO_2_ NTs were not detached from the Ti foil, the XRD pattern showed three diffraction peaks at 35.51°, 40.585°, and 53.404°, which corresponded to the (100), (101), and (102) crystallographic planes of Ti metal (Ti phase COD database (DB) card no. 9016190). The existence of the Ti metal phase in the XRD pattern was in good agreement with the previously published reports [39,40,41]. Some significant diffraction peaks were observed at 25.702°, 37.38°, 38.827°, 48.28°, 54.40°, 55.34°, 63.348°, and 69.24°, which corresponded to the (101), (103), (112), (200), (105), (211), (213), and (116) crystal planes of anatase TiO_2_ (anatase TiO_2_ phase COD database (DB) card no. 1010942). No diffraction peak corresponding to rutile TiO_2_ was observed. Anatase TiO_2_ was preferred as the catalyst support because its properties ensured proper distribution and homogeneity of the catalyst [42].

A small peak was observed at 2θ of 38.32°, which can be assigned to the (111) planes of gold (Au phase COD database (DB) card no. 9011612) [43]. The low peak intensity of Au NPs was in good agreement with the EDX intensity since a small amount of Au NPs were loaded. In addition, no peak of Au-Ti was found, suggesting that both Au NPs and TiO_2_ maintained their native structure and indicated the nanocomposite formation.

### 3.3. Electrochemical Properties of the Au NPs-TiO_2_ NTs Composite Electrode

**Figure 4:**
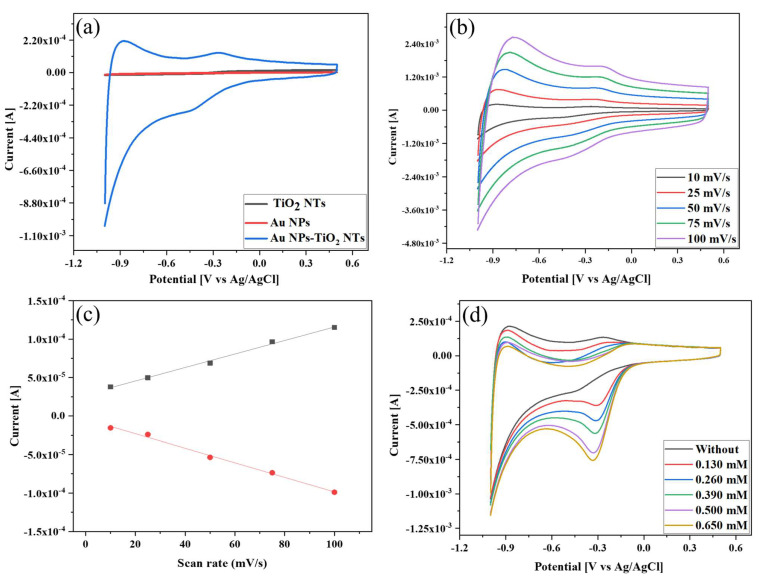
(**a**) Cyclic voltammetry (CV) of different modified electrodes without H_2_O_2_ in 0.1 M PBS (pH = 7.0) at a scan rate of 10 mV/s; (**b**) CV of Au NPs-TiO_2_ NTs composite electrode without H_2_O_2_ in 0.1 M PBS (pH = 7.0) at different scan rates; (**c**) corresponding anodic (black) and cathodic peak current (red) versus scan rate; (**d**) CV of Au NPs-TiO_2_ NTs composite electrode with different H_2_O_2_ in 0.1 M PBS (pH = 7.0) at a scan rate of 10 mV/s.

Figure 4a illustrates the CV of TiO_2_ NTs, Au NPs, and Au NPs-TiO_2_ NTs electrodes in the potential range of −1.0 V to 0.5 V in 0.1 M PBS (pH 7.0) without H_2_O_2_ at a scan rate of 10 mV/s. The Au NPs-TiO_2_ NTs composite electrode exhibited a distinctly enhanced redox peak in comparison with the TiO_2_ NTs and Au NPs electrode. This redox peak indicated the increased electroactive active area and fast electron-transfer behavior of the Au NPs-TiO_2_ NTs composite. This redox peak was formed due to the formation of gold oxide during the forward scan and the subsequent reduction of gold oxide during the reverse scan [39,44]. Alongside this, the reaction kinetics of the composite electrode was investigated by recording the CV responses in 0.1 M PBS (pH 7.0) without H_2_O_2_ at different scan rates, as shown in Figure 4b. It was observed that the oxidation and reduction peak currents increased with increasing scan rate from 10 to 100 mV/s. Figure 4c shows that the anodic and cathodic peak current increase was linear with the scan rate, indicating that the electrochemical reaction was a surface-controlled process.

### 3.4. Electrochemical H_2_O_2_ Sensing of Au NPs-TiO_2_ NTs Composite Electrode

The electrocatalytic activity of Au NPs-TiO_2_ NTs composite electrode toward H_2_O_2_ was examined by adding 0 to 0.650 mM of H_2_O_2_ in 0.1 M PBS (pH 7.0) at a scan rate of 10 mV/s via CV, as shown in Figure 4d. With the continuous increase in the concentration, the reduction peak current gradually increased, and the reduction potential shifted toward negative. This negative shift and the broadening in peak potential with increasing H_2_O_2_ were consistent with a previous report [45]. This top-notch sensing behavior can be attributed to the porous structure of TiO_2_ NTs, which provided a large surface area effective for dispersing or stabilizing Au NPs. The detection mechanism can be expressed as follows [46]:

$$H_{2}O_{2}+e^{-}+\mathrm{Au}↔\mathrm{Au}-{\mathrm{OH}}_{\mathrm{ads}}+{\mathrm{OH}}^{-}$$

$$\mathrm{Au}-\mathrm{OH}\;+e^{-}↔\mathrm{Au}\;+{\;\mathrm{OH}}^{-}$$

$$2{\mathrm{OH}}^{-}+2H^{+}↔\;2H_{2}O$$

### 3.5. Identification of Suitable Experimental Conditions

**Figure 5:**
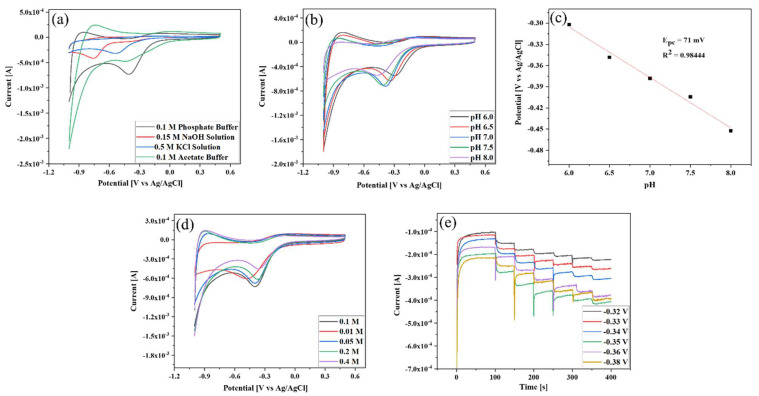
CV-based identification of (**a**) suitable electrolyte media; (**b**) suitable pH; (**c**) corresponding reduction potential vs. pH curve; (**d**) suitable PBS (pH 7) concentration in the presence of 0.5 mM of H_2_O_2_ at a scan rate of 10 mV/s; (**e**) appropriate amperometric reduction potential by adding 60 µM of H_2_O_2_ in 0.1 M PBS (pH 7.0).

Selecting suitable working environments such as an optimal electrolyte solution, pH, buffer concentration, and reduction potential is a prerequisite for sensor development. Figure 5a presents the CV curves of Au NPs-TiO_2_ NTs composite electrode in the presence of 0.5 mM of H_2_O_2_ in different electrolyte media at 10 mV/s. All CV curves exhibited a single peak due to H_2_O_2_ reduction, where the maximum current was achieved in 0.1 M PBS. Hence, 0.1 M PBS was selected, and CV was subsequently run to identify the appropriate pH of 0.1 M PBS. Figure 5b demonstrates that the reduction peak current started to rise with increasing pH from 6.0 to 7.0, after which the reduction current did not increase with increasing pH. The highest reduction peak current was found at pH 7. Thus, pH 7 was selected for this study. Here, the H_2_O_2_ reduction was a pH-dependent reaction because the peak potential shifted to negative as the pH increased. Using this relationship, a potential vs. linear pH graph showed slope and R^2^ values of 71 mV and 0.98444, respectively (Figure 5c). Since the computed slope value was near the predicted Nernst value, the reduction of H_2_O_2_ was a two-electron two-proton reaction [47,48]. In addition, CV was also performed to identify the appropriate PBS concentration in the presence of 0.5 mM H_2_O_2_ at a scan rate of 10 mV/s. Figure 5d shows that 0.1 M PBS gave the maximum reduction current toward H_2_O_2_ compared with other concentrations. Hence, 0.1M PBS was considered.

Identifying the amperometric reduction potential is crucial because the appropriate potential affects sensor performances. Multi-step chronoamperometry was performed to determine the reduction potential by adding 60 μM H_2_O_2_ to 0.1 M PBS (pH 7.0). Figure 5e shows that all six potentials responded to the addition of H_2_O_2_ during the analysis. The rate of current change was low (below −0.35 V), and when it was increased further to −0.36 V and −0.38 V, it still responded less than −0.35 V. Therefore, −0.35 V was selected as the working potential.

### 3.6. Amperometric Detection of H_2_O_2_ on Au NPs-TiO_2_ NTs Composite Electrode

The detection sensitivity of the Au NPs-TiO_2_ NTs composite electrode was studied using multi-step chronoamperometry by adding H_2_O_2_ to 0.1 M PBS at −0.35 V under continuous stirring.

**Figure 6:**
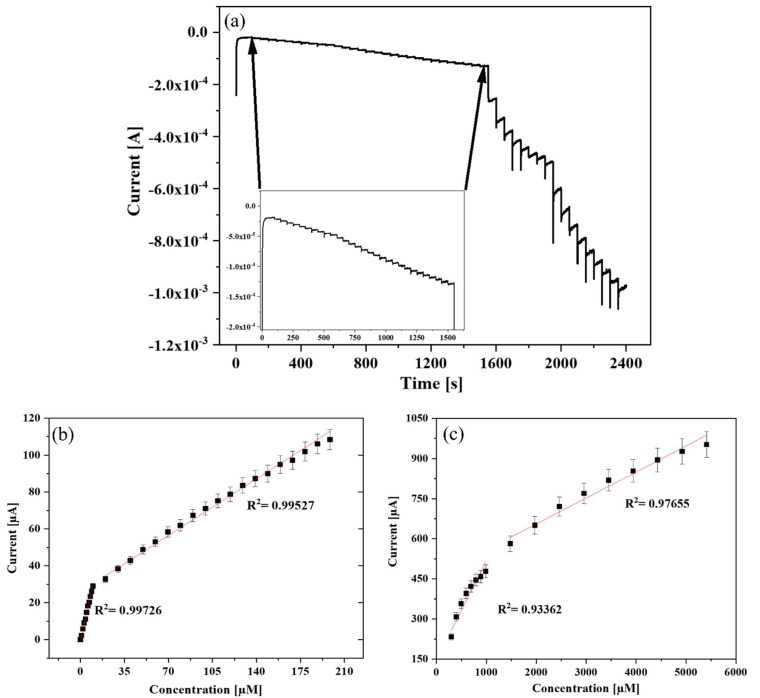
(**a**) Multi-step chronoamperometry of Au NPs-TiO_2_ NTs composite electrode to the successive addition of H_2_O_2_ in 0.1 M PBS (pH 7.0) at −0.35 V. Inset: the magnified view of low concentrations of H_2_O_2_; respective calibration curve of H_2_O_2_ concentration vs. current (**b**) at lower concentrations and (**c**) at higher concentrations.

Figure 6a displays the amperometric current-time (I-t) curves from 1 µM to 5.5 mM of H_2_O_2,_ while the inset displayed curves from 1 to 200 µM. The composite electrode achieved a steady current (95%) within 1.55 s after injection of H_2_O_2_. This speedy response was due to the active role played by the small-sized Au NPs on the electrode surface, which had tiny conduction centers [49]. 

The corresponding current vs. concentration calibration plots are displayed in Figure 6b,c, where all added H_2_O_2_ was linear with the current changes. Four linear ranges were obtained from the calibration plot due to different H_2_O_2_ adsorption and alteration in the electrocatalytic reduction kinetics of H_2_O_2_ on the electrode surface. At low concentrations, the rate-determining step of H_2_O_2_ reduction was dominated by H_2_O_2_ adsorption, while at high concentrations, H_2_O_2_ activation was the dominant determinant. In the middle area, the H_2_O_2_ reduction kinetics was simultaneously mediated by adsorption and activation [50,51]. The fitting curve in Figure 6b exhibits two linear ranges from 1 μM to 9.97806 μM (R^2^ = 0.99726) and from 19.93 μM to 198.47 μM (R^2^ = 0.99527). Another fitting curve is shown in Figure 6c, which exhibits two linear ranges from 297.29 μM to 987.89 μM (R^2^ = 0.93362) and from 1.48 mM to 5.413 mM (R^2^ = 0.97655). In addition, the sensitivity calculated from the linear curve was found to be 519.38 µA/mM. The limit of detection (LOD) of the sensor was estimated using the standard deviation of blank [52]:

(1) $$LOD=3\frac{s}{b}$$

where b is the calibration curve slope and s is the standard deviation of blank current. The detection limit was calculated to be 104.4 nM. The analytical performance of the developed H_2_O_2_ sensor was superior to or comparable with many previously advanced catalysts and even HRP and Hb-based sensors (Table 1).

### 3.7. Selectivity, Reproducibility, and Repeatability Study

**Figure 7:**
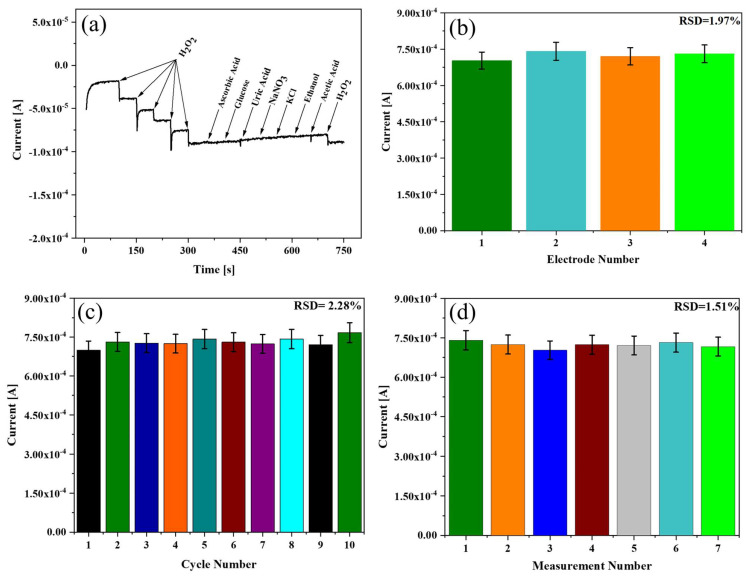
(**a**) Selectivity study of Au NPs-TiO_2_ NTs composite electrode exposed to H_2_O_2_ and ascorbic acid, glucose, uric acid, NaNO_3_, KCl, ethanol, and acetic acid in 0.1 M PBS (pH 7.0) at E = −0.35 V; (**b**) CV-based reproducibility study; repeatability study; (**c**) 10 continuous cycles and (**d**) different measurement times in 0.1 M PBS at 10 mV/s containing 0.5 mM H_2_O_2_.

The effects of intrusive compounds such as ascorbic acid, glucose, uric acid, NaNO_3_, KCl, ethanol, and acetic acid were studied through the amperometric I-t curve technique (Figure 7a). There was a sharp increase in the reduction current after the addition of 10 µM H_2_O_2_. However, after injecting 180 µM of the interfering compounds, no apparent change in the current response was observed that could affect the performance of the sensor. These results indicated the high selectivity of the Au NPs-TiO_2_ NTs composite electrode.

The reproducibility and repeatability of the Au NPs-TiO_2_ NTs composite electrode were explored by CV in the presence of 0.5 mM of H_2_O_2_ in 0.1 M PBS (pH 7.0) at 10 mV/s. The CV of four electrodes prepared under the same conditions (Figure 7b) exhibited almost the same reduction current response with a relative standard deviation (RSD) of 1.97%. The repeatability was explored in two different ways to assess the quality of the sensors. Figure 7c displays the CV curve of ten uninterrupted cycles with a slight fluctuation in the current response, where the RSD value was found to be 2.28%. Further, the repeatability of the electrode was performed in seven successive measurements at different times over 2 days. Figure 7d shows the RSD to be less than 1.51%.

### 3.8. Stability of the Electrode

**Figure 8:**
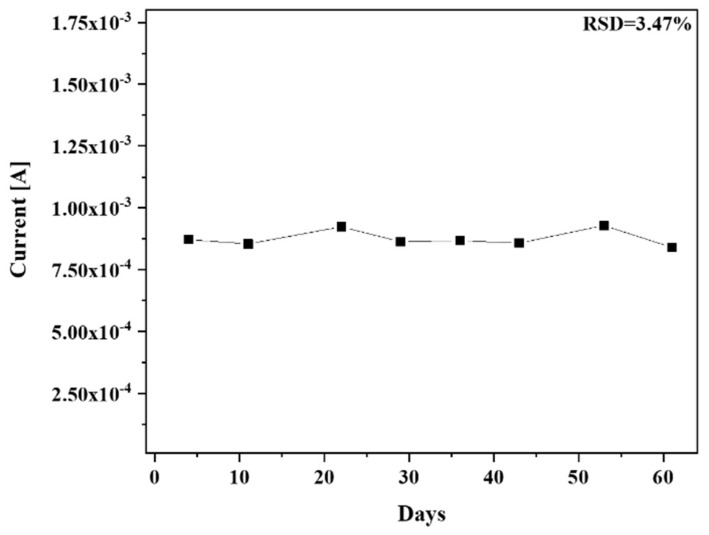
Stability study of Au NPs-TiO_2_ NTs composite electrode with 1 mM of H_2_O_2_ in 0.1 M PBS at 10 mV/s.

To investigate the stability of the Au NPs-TiO_2_ NTs composite electrode, it was stored at room temperature, and CV was performed with 1 mM H_2_O_2_ in 0.1 M PBS (pH 7.0) at 10 mV/s. The developed electrode retained 96.4% of its initial current response for H_2_O_2_ up to 61 days with an RSD of 3.47%, as shown in Figure 8.

### 3.9. Real Sample Analysis

**Figure 9:**
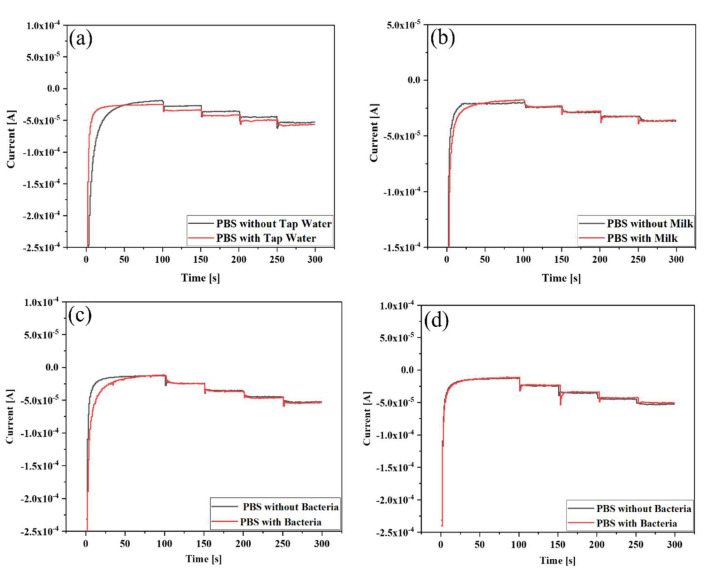
Amperometric responses of the electrode upon stepwise addition of 10 μM H_2_O_2_ in PBS (pH 7) containing (**a**) tap water, (**b**) milk, (**c**) *L. plantarum* from *tapai*, and (**d**) *L. plantarum* from pickle.

The H_2_O_2_-sensing performance of the Au NPs-TiO_2_ NTs composite electrode was evaluated using tap water, milk, and bacteria through multi-step chronoamperometry. During analysis, 10 µM H_2_O_2_ was injected every 50 s into the real samples containing 0.1 M PBS. The results are presented in Figure 9a, and Table 2 shows good recovery of H_2_O_2_, ranging from 109.72% to 100.62%.

The H_2_O_2_ sensing results in milk are shown in Figure 9b and Table 3. The sensor displayed perfect consistency during detection, where the recovery was from 98.33% to 111.15%. The concentration of H_2_O_2_ used in milk was lower than the H_2_O_2_ limit (14.7 μM) set by the US Food and Drug Administration (FDA) for food packaging [69].

The sensing performance on a real sample of Au NPs-TiO_2_ NTs composite electrode was also evaluated using *L. plantarum* bacteria from two different sources, namely *tapai* and pickle. Figure 9c displays the H_2_O_2_ sensing results in *L. plantarum* from *tapai*. The data tabulated in Table 4 demonstrate a satisfactory recovery in the range of 96.20% to 113.83%. Figure 9d displays the H_2_O_2_-sensing performance of the sensor on *L. plantarum* obtained from pickles, where the recovery range was from 95.10% to 111.63% (Table 5).

The current response did not fluctuate much after the addition of the real samples, indicating that the conductivity and resistance of the electrode did not change much after the addition of the real samples. The analysis of four different samples exhibited almost equivalent recovery percentages, thus suggesting the sensor’s versatility. Finally, it can be said that TiO_2_ NTs support efficiently facilitated the electron transfer of Au NPs and retained the catalytic activity for an extended period, resulting in good sensing performances. The comparison of experimental results (Table 1) and efficient practicality for detecting H_2_O_2_ in real samples suggested their potential use for food quality monitoring.

## 4. Conclusions

In summary, a porous TiO_2_ NTs-supported Au NPs-based nonenzymatic H_2_O_2_ sensor was developed via a simple drop-casting method. The aggregation of small-sized Au NPs was prevented by trapping them in porous TiO_2_ NTs, which played a key role in accelerating the detection sensitivity and stability of the sensor. Consequently, the developed sensor exhibited higher sensitivity, selectivity, stability, wide linearity, and nanomolar LOD over their enzymatic counterparts. Furthermore, the satisfactory recovery of H_2_O_2_ in tap water, milk, and *L. plantarum* bacteria by this Au NPs- TiO_2_ NTs composite sensor indicated its potential as a nonenzymatic sensor.

## Figures and Tables

**Table 1:** Comparison of sensor performance.

Electrode Materials	Linear Range	Detection Limit	Stability (Days)	Ref.
**Au NPs-TiO**_**2**_ ** NTs composite**	1–198.47 μM * 297.29–5413 μM *	**104.4 nM**	**61**	**Current study**
NF-Hb-Cys-Au NPs-SPCE	3–240 μM	0.6 µM	30	[52]
Au nanodots/SS electrode	10 µM–1000 µM	3.97 μM	7	[53]
Fe_3_O_4_–MWCNT ink	0.001–2 mM	0.5 µM	21	[54]
Pth-CuO/GCE	20–3300 μM	3.86 μM	15	[55]
GCE/Ni-Co ABDC	0–7 mM	0.18 mM	16	[56]
Au@TiO_2_/MWCNTs/GCE	5–200 µM and 200 µM–6 mM	1.4 μM	50	[57]
Cu-Cu_2_O/BPC-1	1–2830 μM 2830–8330 μM	0.35 μM	30	[58]
Co_3_O_4_/NiCo_2_O_4_	0.05–41.7 mM	0.2578 µM	9	[59]
4 nm PtNPs/GCE	0.025–0.75 mM	10 µM	10	[60]
GCE-Ag_(paste)_-LDH	125–3200 μM	85 µM	5	[61]
Pt_50_Pd_50_ aerogel	5.1–3190 μM	2.21 μM	6	[62]
MWCNTs-FeC/SPCEs	1–1000 μM	0.49 μM	10	[63]
WS_2_/GCE	10–90 µM	0.88 µM	14	[64]
Au NPs-CNTs/3DF	1–296 μM	1.06 μM	21	[65]
Au-Cu/SPCE	0.05–10 mM	10.93 μM	28	[66]
Au NPs-NH_2_/Cu-MOF/GCE	5–850 μM	1.2 μM	7	[67]
HRP/ß-CD/GCE	1–15 μM	0.4 μM	15	[68]

* Different H_2_O_2_ adsorption and alteration in the electrocatalytic reduction kinetics of H_2_O_2_ on the electrode surface. ABDC: 2-aminobenzene-1,4-dicarboxylic acid, BPC: biomass porous carbon, CD: cyclodextrin, CNTs: carbon nanotubes, Co_3_O_4_: cobalt(II,III) oxide, Cu: copper, Cu_2_O: copper(I) oxide, Cys: cysteamine, Fe_3_O_4_: Iron(II,III) oxide, GCE: glassy carbon electrode, Hb: hemoglobin, HRP: horseradish peroxidase, LDH: layered double hydroxide, MOF: metal organic framework, MWCNTs: multi-walled carbon nanotubes, Ni: Nickle, NiCo_2_O_4_: Nickel cobaltite, Pth: parathormone, SPCE: screen printed carbon electrode, SS: stainless steel, WS_2_: Tungsten disulphide.

**Table 2:** Recovery calculation of H_2_O_2_ in tap water.

Addition No.	H_2_O_2_ Added (μM)	H_2_O_2_ Found (μM)	Recovery (%)
1	9.996	10.968	109.72
2	19.988	20.984	104.98
3	29.976	30.887	103.03
4	39.96	40.211	100.62

**Table 3:** Recovery calculation of H_2_O_2_ in full-cream milk.

Addition No.	H_2_O_2_ Added (μM)	H_2_O_2_ Found (μM)	Recovery (%)
1	9.996	9.829	98.33
2	19.988	20.742	103.77
3	29.976	33.321	111.15
4	39.96	41.93	104.93

**Table 4:** Recovery calculation of H_2_O_2_ in *L. plantarum* bacteria from *tapai*.

Addition No.	H_2_O_2_ Added (μM)	H_2_O_2_ Found (μM)	Recovery (%)
1	10.01	9.63	96.20
2	20.02	22.65	113.13
3	30.00	33.25	110.83
4	39.99	41.66	104.17

**Table 5:** Recovery calculation of H_2_O_2_ in *L. plantarum* bacteria from pickle.

Addition No.	H_2_O_2_ Added (μM)	H_2_O_2_ Found (μM)	Recovery (%)
1	10.01	9.52	95.10
2	20.02	22.08	110.28
3	30.00	33.49	111.63
4	39.99	42.91	107.30

## Data Availability

Not applicable.
